# Coal burning-derived SO_2_ and traffic-derived NO_2_ are associated with persistent cough and current wheezing symptoms among schoolchildren in Ulaanbaatar, Mongolia

**DOI:** 10.1186/s12199-019-0817-5

**Published:** 2019-11-27

**Authors:** Dambajamts Enkh-Undraa, Seiji Kanda, Masayuki Shima, Takaki Shimono, Mari Miyake, Yoshiko Yoda, Saijaa Nagnii, Toshimasa Nishiyama

**Affiliations:** 1 Department of Hygiene and Public Health, Kansai Medical University, 2-5-1 Shinmachi, Hirakata, Osaka Prefecture Japan; 2 Department of Public Health, Hyogo College of Medicine, 1-1 Mukogawacho, Nishinomya, Hyogo Prefecture Japan; 3 Environmental Health Research Center, Public Health Institute, Enkhtaiwan Avenue 17, 3rd Khoroo Bayanzurkh, Ulaanbaatar, Mongolia

**Keywords:** Air pollution, SO_2_ and NO_2_, Respiratory symptoms, Schoolchildren

## Abstract

**Background:**

Children in Ulaanbaatar are exposed to air pollution, but few epidemiological studies have been conducted on the effects of environmental risk factors on children’s health. Also, no studies have yet examined the prevalence of respiratory symptoms in children in suburban areas, where air quality-monitoring stations have not yet been installed. This cross-sectional study evaluated the associations between outdoor air pollution and respiratory symptoms among schoolchildren in urban and suburban districts of Ulaanbaatar.

**Methods:**

The ATS-DLD-78 C questionnaire was used to investigate the respiratory symptoms of schoolchildren aged 6–12 years (*n* = 1190) who lived in one of three urban districts or a suburban district of Ulaanbaatar. In each district, the outdoor concentrations of nitrogen dioxide (NO_2_) and sulfur dioxide (SO_2_) were measured at two sites (at ≤100 m and > 100 m from the nearest major road) in the 2-year period from 2015 to 2016. The associations between health outcomes and exposure to air pollutants were estimated using the multinomial logistic regression method.

**Results:**

The outdoor concentration of SO_2_ was significantly associated with persistent cough symptom (OR = 1.12, 95% CI 1.04–1.22). Furthermore, the outdoor concentration of NO_2_ was significantly associated with the current wheezing symptom (OR = 1.33, 95% CI 1.01–1.75) among children in urban and suburban.

**Conclusions:**

The prevalence of persistent cough symptom was markedly high among the schoolchildren in urban/suburban districts of Ulaanbaatar. Overall, the increases in the prevalence of respiratory symptoms among children might be associated with ambient air pollution in Ulaanbaatar.

## Background

Ulaanbaatar is the capital city of Mongolia, and it is located in a basin surrounded by four mountains. Its geographical location and cold climate make it highly susceptible to environmental pollution. For example, temperature inversion layers can trap cold air in such basins, which makes it less likely that air pollutants will diffuse [1]. The current population of Ulaanbaatar is 1.3 million, which represents >  45% of Mongolia’s population. Ulaanbaatar has a relatively young population, with approximately 30.1% of the total population aged < 15 years [2].

Currently, Ulaanbaatar is one of the most polluted cities in the world [3–5]. The city has expanded considerably due to internal migration, and much of the population lives on the outskirts of the city in traditional nomadic dwellings (gers). In “gers”, raw coal is still used for heating and cooking, and the mean amounts of coal and wood used per year in Ulaanbaatar are 5 tons and 3 m^3^, respectively [6]. Significant internal immigration from rural areas of Mongolia to Ulaanbaatar has occurred in recent decades [2]. Urbanization has played a major role in increasing air pollution in the city, as it has resulted in population growth, the expansion of areas containing lots of “gers”, and marked increases in the number of vehicles and traffic jams. The number of vehicles has increased rapidly from 75,000 vehicles in 2005 to 300,000 vehicles in 2014 [6]. Significant effects of air pollution and seasonality on health-related quality of life have been detected in adult patients with bronchial asthma, and the morbidity rate of respiratory diseases increased by 44.8% from 2004 to 2008 [7] . Also, 9.7% of total deaths, 29% of cardiopulmonary deaths, and 40% of lung cancer deaths in Ulaanbaatar in 2009 were caused by air pollution [4].

Various adverse effects of outdoor air pollution on children’s respiratory health have been identified. It has been reported that exposure to air pollutants significantly increases the incidence of respiratory symptoms in children [8–12]. Mongolian children are more exposed to air pollution than children living in other countries. However, few epidemiological studies on the effects of environmental risk factors on children’s health in Mongolia have been conducted [13]. Previous studies found correlations between ambient air pollutant levels and spontaneous abortion in Ulaanbaatar [14]. Furthermore, the increased concentrations of sulfur dioxide (SO_2_) and nitrogen dioxide (NO_2_) have been reported to be associated with lower baby weights [15]. Mongolian children’s urinary 1-hydroxypyrene (1-OHP) levels were reported to be associated with the ambient air concentrations of the polycyclic aromatic hydrocarbon co-pollutants SO_2_ and NO_2_, and it was suggested that the increased urinary 1-OHP levels exhibited by Mongolian children might be attributed to PAH emissions from coal burning and traffic [16]. Regarding the respiratory health effects of urban and rural environments on children, changes in the concentrations of air pollutants and the household use of solid fuel have been found to be associated with fatal or non-fatal respiratory diseases, airway narrowing, or a slower rate of forced vital capacity growth [17, 18].

Therefore, this study evaluated the associations between outdoor air pollution and respiratory symptoms among schoolchildren in urban and suburban areas of Ulaanbaatar.

## Methods

### Study design

We used a cross-sectional study design to investigate the effects of short-term outdoor exposure to NO_2_ and SO_2_ on the respiratory symptoms of children during the 2-year period from 2015 to 2016.

### Participants and residential areas

**Fig. 1:**
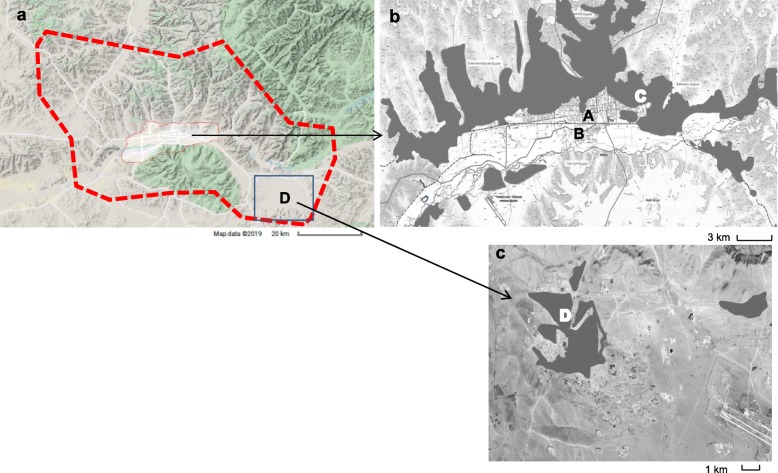
**a** Map of Ulaanbaatar. **b** The locations of the urban study sites; A, B: apartments (white), C: “gers” (black). **c** The location of the suburban site; D: apartments (white), “gers” (black)

The participants were 1200 children (age 6–12 years) from five public elementary schools. The schools were located in three urban districts (Sukhbaatar, Khan-Uul, and Bayanzurkh) and a suburban district (Nalaikh) in Ulaanbaatar. The districts were named A, B, C, and D, respectively. Figure 1 shows the locations of the study areas in Ulaanbaatar. District A is located in the center of the city. Two public schools in district A were included in this study. District B is located adjacent to district A, and industrial activity takes place in this district. District C is located at the eastern end of the urban area, and most residents live in “gers”. For comparative analysis, we selected suburban area D, which is located 40 km southeast of the urban area. It is the nearest suburban area to the capital and has increased rapidly in size, as internal migrants have moved from rural areas; therefore, approximately 70% of the residents live in “gers”.

In each of these districts, the targeted school was close to traffic major road within 200 m. In the urban areas, outdoor air pollutant levels were measured by ambient air quality-monitoring stations. However, such systems have not yet been installed in district D (the suburban area; Fig. 1).

### Distance from each subject’s residence to the nearest major road and traffic volume

We estimated the distance between each subject’s residence and the nearest major road using the GeoNET 9.7.107 GPS navigator for mobile mapping software. Then, we categorized the subjects into ≤ 100  m and > 100 m groups, depending on the distance from their residence to the nearest major road. Also, we measured the daily traffic volume at the major road nearest to each school four times in 2015. The mean daily traffic volume was determined using a vehicle counter (Tempomat CRM, IV, Radarlux).

### Questionnaire

The survey about respiratory symptoms was based on a questionnaire adapted from the American Thoracic Society Epidemiologic Standardization Project questionnaire [19]; i.e., the ATS DLD 78 C questionnaire for children, which has been used in previous studies [8, 20–24]. The questionnaire was translated into Mongolian and then distributed to the participants via their schools. It was completed by their parents at home after obtaining written parental consent. The participants were given a week to return the questionnaire. All of the questionnaires were given ID numbers prior to the analysis.

The items included in the questionnaire were age, sex, residential address, whether the subject was bottle-fed in infancy, how long they had lived at their current residence, the heating system used in their home, their parents’ smoking habits at home, and their history of respiratory disease and current respiratory symptom status. The questionnaire covered the following respiratory symptoms: persistent cough, persistent phlegm, current wheezing, and asthma-like symptom. Respiratory symptoms were recorded as being present if a participant answered, “Yes”, to a relevant question. The participants were provided with explanations about each respiratory symptom [see Additional file 1: Table S1].

### Passive sampling of air pollutants near schools

We selected NO_2_ and SO_2_ as indicators of outdoor air pollution. The ambient air concentrations of NO_2_ and SO_2_ were measured near the schools. The concentrations of these pollutants were measured in February, April, August, and November in 2015 and in January, April, July, and October in 2016. The two field blanks used for the concentration analysis and the filters for NO_2_ and SO_2_ were exchanged five times each at intervals of approximately 48 h. The samplers were installed for 10 days (14,400 ± 5 min). We measured the mean air temperature and relative humidity during the same period as the NO_2_ and SO_2_ measurements were obtained. The levels of NO_2_ and SO_2_ were assessed using co-located Ogawa passive samplers (Ogawa & Co., Ltd., Kobe, Japan), which were used in previous studies [4, 25–28]. The passive samplers were installed at two sites, which were located ≤ 100 m and > 100 m from the nearest major road, respectively, at a height of approximately 2.5 m.

### Statistical analyses

All statistical analyses were performed using the PASW Statistics software, v.18 (Chicago, IL, USA). All questionnaire data were analyzed based on the ID numbers assigned to the questionnaires. The associations between respiratory symptoms and personal and environmental factors were analyzed statistically via multinomial logistic regression analysis. The respiratory symptoms were used as dependent variables, and potential confounding factors were used as independent variables. The following variables were considered to be potential confounding factors: gender, age, how long the subject had lived at their current residence (years), history of asthma diagnosed by a doctor, history of allergies or respiratory disease before the age of 2 years, history of pneumonia, being bottle-fed in infancy, parental smoking habits, and the distance between the subject’s residence and the nearest major road. We also analyzed the associations between the concentrations of air pollutants and respiratory symptoms; i.e., logistic regression was used to calculate odds ratio (OR) and 95% confidence interval (CI) values. *P*-values of <  0.05 (two-tailed) were considered statistically significant. Adjusted OR were estimated and scaled to represent a 1-ppb increase in the NO_2_ or SO_2_ concentration, based on the mean 2-year concentrations and traffic volume data for each district. The paired *t*-test was used to examine the significance of differences in the seasonal concentrations of SO_2_ and NO_2_, and changes in the NO_2_ concentration due to the distance from the main road. We evaluated the statistical significance of differences in the frequency of respiratory symptoms between the residential areas using one-way analysis of variance (ANOVA). *P*-values of <  0.05 (two-tailed) were considered to be statistically significant.

### Analytical method for NO_2_

The NO_2_ collection filters (14.5 mmφ) were placed in 25-ml glass vials. Then, 8 ml distilled water was added to the filter-containing vials, and each vial was subjected to shaking for extraction. After 30 min of extraction, the vials were refrigerated at 4–5 °C for 30 min. Then, 2 ml of sulfanilamide solution and 1-naphthyl-ethylenediamine dihydrochloride (NEDA; 0.56 g dissolved in 100 ml distilled water, stored at − 4 °C) solution were added in a 10:1 ratio. The resultant mixture was simultaneously shaken and cooled for 30 min. After that, the vials were returned to room temperature. The absorbance of the samples was determined with a spectrophotometer at a wavelength of 545 nm.

### Analytical method for SO_2_

Filter papers were put in 25-ml glass vials containing 8 ml distilled water. Then, the vials were shaken for extraction for about 30 min, before the filter papers were collected. Next, hydrogen peroxide solution (H_2_O_2_, 1.75%) was added, and the vials were shaken slowly for 10 min, before the mixture was equilibrated at room temperature and analyzed using ion chromatography. SO_2_ concentrations were analyzed via ion chromatography at the laboratory of Ogawa & Co., Ltd. (Kobe, Japan). The same procedure was used for all blank samples and standard solutions. We calculated the ambient concentrations of NO_2_ and SO_2_ using the following equations:

$$ {\mathrm{NO}}_2={\upalpha}_{\mathrm{NO}2}\times {W}_{\mathrm{NO}2}/\mathrm{t} $$

$$ {\mathrm{SO}}_2={\upalpha}_{\mathrm{SO}2}\times {W}_{\mathrm{SO}2}/\mathrm{t} $$

α_NO2,_ α_SO2_: ppb concentration conversion coefficient (ppb min/ng).

*W*_NO2_: quantity (ng) collected in NO_2_ collection elements.

*W*_SO2_: quantity (ng) collected in SO_2_ collection elements.

*T*: exposure time (min).

α_NO2_ was calculated based on the temperature, humidity, and exposure time during the measurement period. We used meteorological data from the private Weather Underground Station Network, which directly collects data from weather stations around the world [29]. The abovementioned analytical methods for NO_2_ and SO_2_ concentrations were based on the Ogawa protocol.

## Results

### Pollutant exposure assessment

During the measurement period, the mean daily temperatures varied among the seasons. In winter (February 2015 and January 2016), spring (April 2015 and 2016), summer (August 2015 and July 2016), and fall (November 2015 and October 2016), the mean daily temperature was − 22.1 °C, 8.5 °C, 22.0 °C, and − 8.2 °C and the mean relative humidity value was 65%, 35%, 52%, and 54%, respectively. Table 1 summarizes the distribution of mean air pollutant concentrations in the four districts of Ulaanbaatar during the 2-year period from 2015 to 2016. In each study area, the concentrations of SO_2_ and NO_2_ exceeded the ambient air quality standard values for Mongolia [annual mean concentration of SO_2_: 10 μg/m^3^ (3.8 ppb), annual mean concentration of NO_2_: 30 μg/m^3^ (16 ppb); MNS 4585:2007]. In districts A–D, the 2-year mean concentrations of SO_2_ were 5.2 ppb, 4.4 ppb, 10.1 ppb, and 7.1 ppb, respectively, and the maximum concentrations of SO_2_ were 36 ppb, 39 ppb, 63 ppb, and 46 ppb, respectively. The minimum concentration of SO_2_ was 0.0 ppb in each district. Higher SO_2_ concentrations were detected in districts C and D than in districts A and B.

**Table 1:** Distribution of SO_2_ and NO_2_ concentrations in 2-year average among 4 districts in Ulaanbaatar, 2015–2016

	Mean ± SD	Min	25	50	75	Max
District/SO_2_ (ppb)						
A	5.2 ± 6.7	0.0	0.8	2.6	2.6	36.0
B	4.4 ± 6.3	0.0	1.2	2.9	5.5	39.0
C	10.1 ± 14.4	0.0	1.1	3.2	14.5	63.0
D	7.1 ± 9.2	0.0	0.6	3.8	11.0	46.0
District/NO_2_ (ppb)						
A	30.0 ± 17.9	12.0	18.0	24.0	36.8	86.0
B	27.7 ± 15.4	7.0	17.0	23.0	33.8	74.0
C	30.0 ± 20.2	4.0	17.0	23.0	36.8	99.0
D	18.2 ± 15.8	3.0	8.0	10.5	26.0	68.0

*SO*_*2*:_sulfur dioxide, *NO*_*2*_: nitrogen dioxide, *ppb*: parts per billion, *SD*: standard deviation, min-minimum, maxi-maximum values, air quality standard of annual

Average concentration SO_2_: 10 μg/m^3^ (3.8 ppb), NO_2_: 30 μg/m^3^ (16 ppb)

**Fig. 2:**
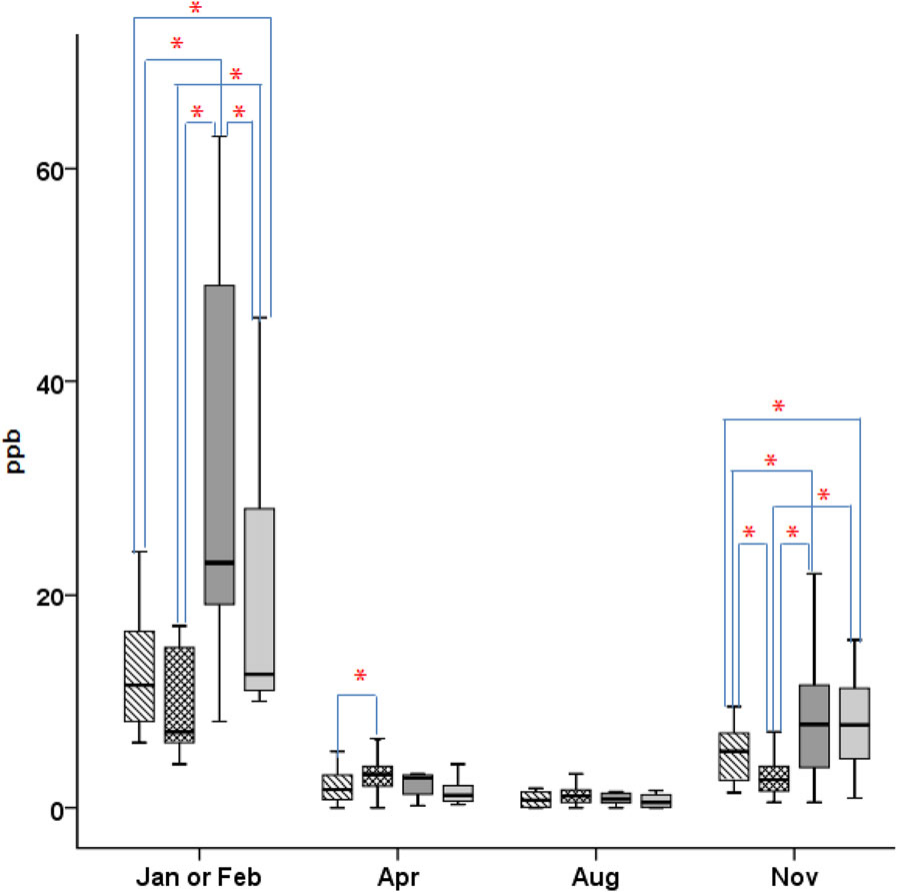
Comparison of the outdoor SO_2_ concentrations in each district in each season. District A: box with stripes, district B: mesh box, district C: black box, district D: gray box; Asterisk indicates statistically significant (*p* < 0.05); air quality standard for the mean annual SO_2_ concentration: 10 μg/m^3^ (3.8 ppb)

For NO_2_, the mean annual concentration was 30.0 ppb, 27.7 ppb, 30.0 ppb, and 18.2 ppb in districts A–D, respectively, and the maximum concentrations were 86 ppb, 74 ppb, 99 ppb, and 68 ppb, respectively. Higher NO_2_ concentration were detected in districts A, B, and C than in district D. Figure 2 shows seasonal comparisons of outdoor SO_2_ concentrations between the four districts. High SO_2_ concentrations were detected in each district in January and February. During this period, the mean SO_2_ concentration was 14.3 ± 9.0 ppb, 12.3 ± 10.2 ppb, 21.9 ± 10.3 ppb, and 19.6 ± 11.6 ppb in districts A–D, respectively. There were significant differences in the SO_2_ concentration between each district, except between districts A and B (*p* < 0.01) [see Additional file 1: Table S3]. In April, the mean SO_2_ concentrations in each district were roughly similar (the mean SO_2_ concentrations of districts A–D decreased to 2.0 ± 1.5 ppb, 3.0 ± 1.9 ppb, 2.6 ± 1.8 ppb, and 2.1 ± 3.2 ppb, respectively). Only the SO_2_ concentrations of districts A and B differed significantly (*p* < 0.05). In August, the mean SO_2_ concentration decreased to 1.0 ppb in each district. However, in November, the mean SO_2_ concentration increased again to 5.0 ± 2.5 ppb, 2.9 ± 1.7 ppb, 9.6 ± 8.3 ppb, and 8.0 ± 4.6 ppb in districts A–D, respectively. There were significant differences between the SO_2_ concentrations of all districts, except for between the SO_2_ concentrations of districts C and D.

**Fig. 3:**
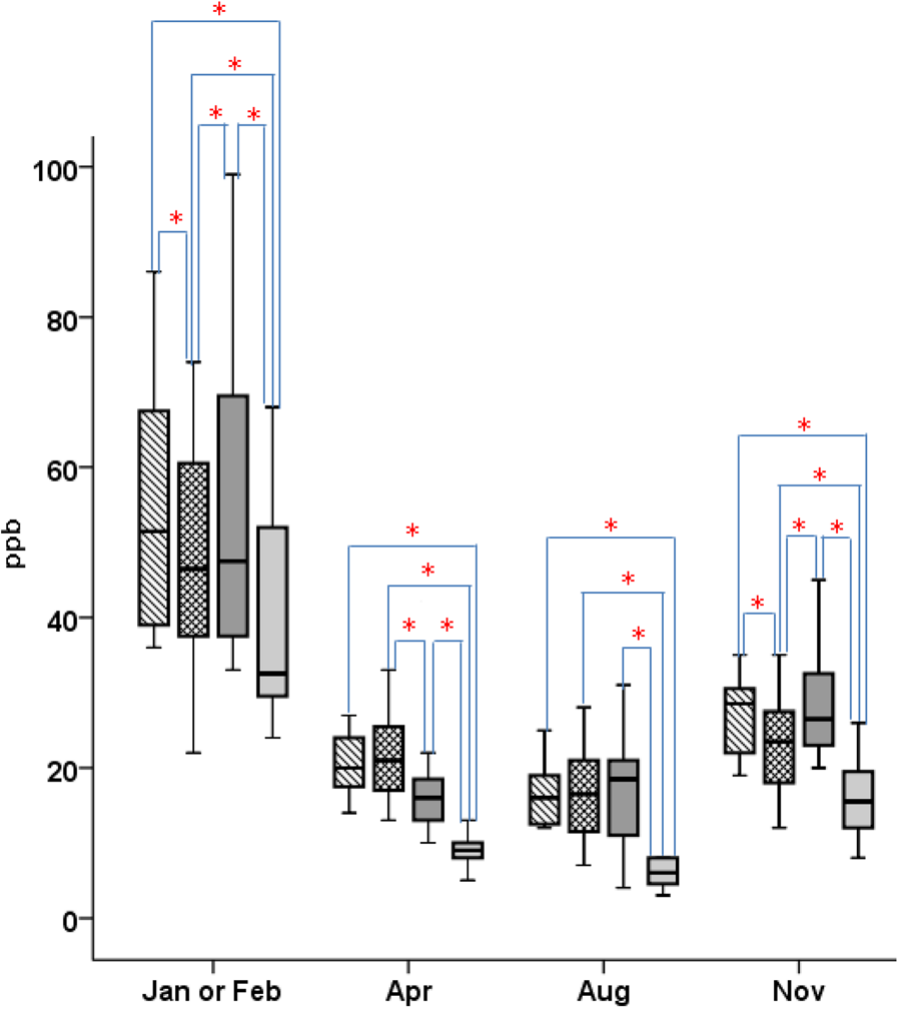
Comparison of the outdoor NO_2_ concentrations in each district in each season. District A: box with stripes, district B: mesh box, district C: black box, district D: gray box; Asterisk indicates statistically significant (*p* < 0.05); air quality standard for the mean annual. NO_2_ concentration: 30 μg/m^3^ (16 ppb)

The results indicated that in all districts, the mean NO_2_ concentration exceeded the ambient air quality standard value in all months, except for August. Figure 3 shows a seasonal comparison of outdoor NO_2_ concentrations between the districts. The concentration of NO_2_ was high in each district in February, especially in the urban districts (A, B, and C). The mean NO_2_ concentration was 54.6 ± 17.1 ppb, 48.1 ± 15.1 ppb, 56.3 ± 21.4 ppb, and 40.5 ± 14.1 ppb in districts A–D, respectively. There were significant differences between the NO_2_ concentrations of all districts, except for between those of districts A and C (*p* < 0.01) [see Additional file 1: Table S4].

In April, the mean NO_2_ concentration decreased to 20.0 ± 3.9 ppb, 21.6 ± 5.4 ppb, 17.5 ± 7.3 ppb, and 9.1 ± 1.9 ppb in districts A–D, respectively. There were significant differences between the NO_2_ concentrations of each district except for between those for districts A and B, and districts A and C (*p* < 0.01). In August, the mean NO_2_ concentrations of the three urban districts were roughly similar and had decreased to 16.5 ± 3.8 ppb, 16.5 ± 6.0 ppb, 16.9 ± 7.4 ppb, and 6.1 ± 1.8 ppb, respectively. The mean NO_2_ concentration of district D differed significantly from those of districts A, B, and C (*p* < 0.000). However, the mean NO_2_ concentration of each district increased again in November. The mean NO_2_ concentration was 28.7 ± 8.5 ppb, 24.7 ± 9.2 ppb, 28.7 ± 8.0 ppb, and 17.4 ± 8.1 ppb in districts A–D, respectively. There were significant differences between the mean NO_2_ concentrations of all districts, except between those of districts A and C (*p* < 0.01). In all districts, the mean NO_2_ concentration in each season exceeded the ambient air quality standard value (except for the NO_2_ concentrations of district D in April and August, Fig. 3).

In addition, in each district we examined the NO_2_ concentrations detected at sites located ≤ 100 m and > 100 m from the nearest major road (hereafter referred to as the ≤ 100 m and > 100 m sites). Table 2 shows the differences in the NO_2_ concentration depending on the distance from the nearest major road. In districts A, B, and C, significantly higher NO_2_ concentrations were detected at the ≤ 100 m sites than at the > 100 m sites. The mean values obtained for the ≤ 100 m and > 100 m sites in each district were as follows: district A, 31.8 ppb vs. 29.1 ppb (*p* < 0.05); district B, 34 ppb vs. 23 ppb (*p* < 0.01); and district C, 33 ppb vs. 27 ppb (*p* < 0.01), respectively. However, there were no significant differences between the values obtained for the ≤ 100 m and > 100 m sites in the suburban district (district D) (17.5 ppb vs. 17.8 ppb; *p* < 0.15).

**Table 2:** The difference in concentration of NO_2_ due to a distance from traffic major road

Distance	≤ 100 m	> 100 m	*p* value
District	NO_2_ (ppb) mean ± SD		
A	31.8 ± 17.6	29.1 ± 19.2	0.026
B	33.5 ± 17.6	23.1 ± 13.5	0.001
C	33.3 ± 21.6	26.3 ± 18.9	0.005
D	17.5 ± 12.2	17.8 ± 13.6	0.147

*NO*_*2*_: nitrogen dioxide, *ppb*: parts per billion, *SD*: standard deviation

≤ 100 m—within 100 m from the traffic major road, > 100 m—outside, *p* value (two-sided)—significance

Mapping software was used to determine the location of each subject’s residence based on their home address, and the distance between each subject’s home and the nearest major road was manually determined for all participants [see Additional file 1: Figure S1]. All elementary schools were located within 200 m of the nearest major road. In the urban districts (A, B, and C), the examined major roads had 2–3 times higher traffic volumes than the major road in the suburban district. The mean ± standard deviation daily traffic volume was 16,842 ± 2211, 16,985 ± 1788, 19,473 ± 2686, and 7187 ± 736 vehicles in districts A–D, respectively.

### Prevalence of respiratory symptoms among children

The survey was carried out in 2015. The questionnaire was completed by 1130 children, and 21 children were excluded because they were living in other districts or did not fill out more than half of the questionnaire. Table 3 shows the personal and environmental data obtained via the questionnaire alongside the prevalence of respiratory symptoms by district. The ATS DLD questionnaire was correctly completed by 1109 (92.4%) participants. Among the subjects aged 6–12 years, the most common respiratory symptom was persistent cough (28.5%). In addition, 20.2% of the subjects reported persistent phlegm, 5.8% had current wheezing, and 0.7% reported asthma-like symptom. Furthermore, the following findings were obtained regarding the frequency of other background factors: being bottle-fed in infancy: 18.4%, a history of respiratory disease before the age of 2 years: 26%, a history of diagnosed asthma: 2.4%, a history of diagnosed pneumonia: 6.2%, history of residence year for > 3 years: 65.6%, parents smoke at home: 43.1%, coal being used for heating at home: 33.5%, and living ≤ 100 m from the nearest major road: 37.4%. In particular, in districts C and D, high prevalence of subjects had persistent cough (32% and 37%, respectively). However, the prevalence of asthma-like symptoms was low.

**Table 3:** Descriptive characteristics of the subjects and the prevalence of respiratory symptoms based on the questionnaire

Residential area	Apartment				Ger dwelling				Total	
District	A urban		B urban		C urban		D suburban			
	*n*	%	*n*	%	*n*	%	*n*	%	*n*	%
Response rate	238	79	273	91	300	100	298	99	1109	92.4
Sex										
Female	129	54	123	45	122	41	141	47	594	53.6
Male	109	46	150	55	178	59	157	53	515	46.4
Age										
6–8 years	135	57	161	59	146	49	176	59	618	55.7
9–12 years	103	43	112	41	154	51	122	41	491	44.3
Feeding in infancy (bottle milk)	52	22	47	17	57	19	48	16	204	18.4
History of respiratory diseases before 2 years old	68	29	89	33	65	22	66	22	288	26.0
History of diagnosed asthma	12	5.0	2	0.7	6	2.0	7	2.3	27	2.4
History of allergic diseases	52	22	57	21	38	12	29	10	176	15.9
History of pneumonia	16	7	25	9	16	5	12	4	69	6.2
Parental smoking habit at home	87	37	103	38	146	49	142	48	478	43.1
History of residence years	150	63	170	62	187	62	220	74	727	65.6
Heating type (coal using)	6	3	11	4	141	47	214	72	372	33.5
Distance from major road < 100 m	119	50	126	46	99	33	69	23	413	37.4
Daily traffic volume	16,842		16,985		19,473		7187		60,487	
Respiratory symptoms										
Persistent cough	60	25	50	18	95	32	111	37	316	28.5
Persistent phlegm	50	21	50	18	63	21	61	21	224	20.2
Asthma-like symptom	4	2	0	0	2	1	2	1	8	0.7
Current wheezing	22	9	14	5	17	6	11	4	64	5.8

### Associations between exposure to outdoor air pollution and respiratory symptoms

Table 4 shows the associations between the outdoor SO_2_ or NO_2_ concentration and the prevalence of respiratory symptoms identified using a logistic regression model adjusted for confounding variables (*n* = 1109). Persistent cough was associated with the outdoor concentration of SO_2_ (OR 1.12, 95% CI 1.04–1.22; per 1 ppb increase), whereas current wheezing was associated with the NO_2_ concentration (OR 1.33, 95% CI 1.01–1.75). Conversely, no statistically significant associations were found between the outdoor SO_2_ or NO_2_ concentration and other symptoms. Also, traffic volume was not associated with respiratory symptoms, such as with persistent cough (OR 0.87, 95% CI 0.73–1.03), persistent phlegm (OR 0.95, 95% CI 0.80–1.14), or current wheezing (OR 0.77, 95% CI 0.58–1.03). The associations between personal or environmental factors and respiratory symptoms were examined using multinomial regression analysis. Current wheezing was found to be significantly associated with being bottle-fed in infancy (OR 2.04, 95% CI 1.14–3.66), and persistent phlegm was demonstrated to be associated with a history of respiratory disease before the age of 2 years (OR 1.58, 95% CI 1.10–2.25). Also, a history of pneumonia was shown to be associated with persistent phlegm (OR 1.90, 95% CI 1.07–3.36) and current wheezing (OR 4.01, 95% CI 1.87–8.60) [see Additional file 1: Table S5]. Furthermore, the prevalence of persistent cough was significantly increased among the children that lived within 100 m of a major road (OR 1.86, 95% CI 1.37–2.51). However, we could not analyze the associations between current asthma-like symptom and confounding factors, as the prevalence of this condition was very low.

**Table 4:** The association between outdoor SO_2_ and NO_2_ concentrations and prevalence of respiratory symptoms among schoolchildren in the questionnaire survey using a logistic model, adjusted for confounding variables (*n* = 1109)

	OR for absolute increase of per 1 ppb in air pollutants	
Respiratory symptoms	SO_2_	NO_2_
Persistent cough	**1.12** (1.04–1.22)	1.14 (0.97–1.34)
Persistent phlegm	1.03 (0.94–1.12)	1.07 (0.91–1.27)
Current wheezing	1.06 (0.91–1.24)	**1.33** (1.01–1.75)

Data are presented as OR—odds ratio (95% CI confidence interval)

Confounding variables: gender, age, history residence year, history of asthma and allergies, history of respiratory diseases before 2 years old, history of pneumonia, feeding method in infancy, parental smoking habits, heating type, distance from the major road

Mean concentrations for outdoor SO_2_ of each district 5.2 ppb, 4.4 ppb, 10.1 ppb, and 7.1 ppb, for NO_2—_30.0 ppb, 27.7 ppb, 30.0 ppb, and 18.2 ppb, respectively. And traffic volume was calculated as a value per thousand vehicles a day of each district: 16.84, 16.99, 19.47, and 7.19, respectively

Values in bold indicate statistical significant: *p* < 0.05

## Discussion

In the present study, the mean concentrations of SO_2_ and NO_2_ exceeded the Mongolian ambient air quality standards in each district, excluding the NO_2_ concentration in the suburban district. The concentrations of both pollutants peaked during winter (maximum concentrations: 36 ppb to 63 ppb for SO_2_ and 74 ppb to 99 ppb for NO_2_, Table 1). Ulaanbaatar city is located in a basin. In winter, a phenomenon called a temperature inversion layer can arise in basins, which reduces the diffusion of air pollutants [30]. In a previous study, the annual mean concentration of SO_2_ in an urban area of Ulaanbaatar was reported to be 12.35 ± 14.53 μg/m^3^ (4.71 ppb) [31]. In our study, the mean concentration of SO_2_ in the three urban districts (A, B, and C) was 6.5 ± 10.3 ppb, and district C, which contains many gers dwelling, exhibited the highest mean SO_2_ concentration. Also, the mean SO_2_ concentration was higher in the districts with large numbers of gers (districts C and D; mean SO_2_ concentrations: 10.1 ppb and 7.1 ppb, respectively) than in the areas that contain lots of apartments (districts A and B; mean SO_2_ concentrations: 5.2 ppb and 4.4 ppb, respectively). Previous studies have detected differences in the mean SO_2_ concentration between “gers” and “non-gers” sites in Ulaanbaatar [28, 31]. A 2011 survey reported a higher mean SO_2_ concentration for “gers” areas (46.60 ppb) than for “non-gers” areas (23.35 ppb) [28]. This can be partly explained by the fact that the 2011 census showed that the population of Ulaanbaatar had increased by 300,000 from 2000 to 2008. Simultaneously, the numbers of residents living in “gers” districts have increased rapidly as a result of internal immigration; i.e., from rural areas to the capital city [32]. In 2010, a study reported that in winter the NO_2_ and SO_2_ concentrations in the urban areas of Ulaanbaatar were 10.7 ± 5.8 ppb and 17 ± 11.8 ppb, respectively [4]. In the present study, the mean concentrations of NO_2_ and SO_2_ in the three urban districts (A, B, and C) were 53.0 ± 8.1 ppb and 18.7 ± 15.0 ppb, respectively, in winter. The urban districts (A, B, and C; 30.0 ppb, 27.7 ppb, and 30.0 ppb, respectively) had higher mean NO_2_ concentrations than the suburban district D (18.2 ppb). The concentrations for the urban districts were 2–3-fold higher than those obtained in the abovementioned study. The reason for this is that the number of vehicles in Ulaanbaatar doubled from 2010 to 2015, and the mean vehicle speed on Ulaanbaatar’s major roads has decreased from approximately 40 km/h to 20 km/h [33, 34]. Due to the poor dispersal conditions encountered in winter, it is assumed that vehicle exhaust emissions accumulate within the city in winter.

NO_2_ is used as a marker of traffic-related pollutant because its concentration peaks close to roadways, but decreases to background levels by about 200 m from the nearest major road [25, 35, 36]. A previous study reported that the NO_2_ concentration was approximately 5 ppb higher at the roadside than at other urban sites in Ulaanbaatar [28]. In our study, the difference in the NO_2_ concentration between the roadside and non-roadside sites was about 3–10 ppb in the urban districts (A, B, and C). Also, the NO_2_ concentration was lower at the sites located > 100 m from the nearest major road than at those located ≤ 100 m from the nearest major road. This result is consistent with our findings regarding traffic volume. Traffic volume was significantly higher in the urban districts (A, B, and C) than in the suburban area (district D). The fact that the concentration of NO_2_ decreases away from major traffic, roads can be considered to be due to the effects of automobile exhaust gas [28, 36]. Central urban areas include many bus routes and experience numerous traffic jams. Due to traffic congestion, vehicles have to slow down, which is considered to increase NO_2_ emissions. In winter, the temperature outside is cold, so drivers gradually move forward with their engines on. Currently, no surveys in Mongolia have directly measured exhaust gas emissions from vehicles.

### Respiratory health outcomes

This study confirmed that Mongolian schoolchildren have high prevalence rates of persistent cough (28.5%), persistent phlegm production (20.2%), current wheezing (5.8%), and asthma-like symptoms (0.7%). When the prevalence was examined by district, the prevalence of persistent cough was high in the suburban compared to the urban district which has similar many gers dwellings (37% vs. 32%, respectively). Conversely, the concentration of SO_2_ was higher in district C than in the suburban (district D). The survey indicated that there are several possible reasons for this first, more children in district D (72%) than in district C (47%) lived in a household in which coal was used. So, it is considered that these children were affected by emissions from coal. Another possible reason is that the children in district D had lived in the same place for a longer period than those in district C; i.e., 74% of the children in district D had lived in the same residence place for > 3 years, whereas the equivalent figure for district C was 62% (data shown in Table 3). In a previous study, in which the subjects had a similar age distribution to those in our study, it was concluded that outdoor air pollution is the major source of black carbon (BC) and fine particulate matter (PM_2.5_) exposure for children living in Ulaanbaatar. Also, it was reported that the children living in a district containing many “gers” were exposed to the highest levels of outdoor air pollution [37]. In our study, persistent cough was significantly more common in the areas containing many “gers” (districts C and D) than in the areas containing large numbers of apartments (districts A and B, *p* < 0.001) [see Additional file 1: Table S6]. In particular, the prevalence rate of persistent cough was significantly higher in the suburban district (district D) than in the urban areas (districts A, B, and C, *p* < 0.000). Even in the suburbs, the use of coal has led to a high prevalence of persistent cough in children. We were reported that on an isolated island without major artificial sources of air pollutants, changes in the concentrations of air pollutants acutely influenced the pulmonary function of healthy subjects [38]. On the other hand, the increased prevalence of respiratory symptoms may also affect the burning of solid waste at the open dumpsites in the gers districts. There still has a problem with solid waste, coal ash and other rubbish, and also low-income households burn old tires, plastic bottles [39, 40]. Das B et al. reported that open burning of municipal solid waste is a poorly characterized and frequently underestimated source of air pollution. It is can trigger health impacts such as acute and chronic respiratory disease [41]. Boadi K. O et al. reported also that inadequate solid waste facilities result in indiscriminate burning and burying of solid waste. There is an association between waste burning and the incidence of respiratory health symptoms among children [42].

In the statistical analysis, we confirmed that the prevalence of persistent cough among children was associated with the concentration of SO_2_. Some Chinese studies have reported that children exhibit enhanced sensitivity to the harmful effects of air pollution. In addition, it was confirmed that genetic susceptibility and/or exposure to common environmental factors have a strong influence on respiratory health [8, 43].

In our study, the current wheezing symptom among children associated an increase in NO_2_ concentration (data shown in Table 4). Nicolai T et al.reported that the frequency of current wheezing among children increased with the NO_2_ concentration [44]. Also, in our study, the children who were bottle-fed in infancy displayed an increased prevalence of current wheezing. Other studies have demonstrated that being breastfed was associated with a lower risk of lung function problems and reduced children’s susceptibility to the respiratory effects of pollutants [45, 46].

The prevalence of persistent cough and phlegm were reported to be more common among children in Mongolia than among children in the north and north-eastern cities of China and Thailand, but the frequencies of current wheezing and asthma-like symptom were similar in all of these places [8, 21, 22, 43]. It is considered that other than the geographical features mentioned above and the temperature inversion layer phenomenon that occurs in winter, meteorological features also have a great influence, as Ulaanbaatar has a longer heating period than China; i.e., it lasts for 8 months from mid-September to mid-May [47]. In winter, when coal consumption increases, the concentrations of air pollutants are expected to rise markedly, which might induce respiratory symptoms in children. However, a previous Japanese study found that persistent cough and phlegm were less common among Japanese children than among Mongolian children, although current wheezing and asthma-like symptom were more common among Japanese children [22]. One possible reason for this is that since the 1970s, the atmospheric concentration of SO_2_ in Japan has reduced owing to the introduction of emission-control technologies. On the other hand, NO_2_ concentrations decreased from 1970 to 1985, but increased from 1985 to 1995. NO_2_ concentrations are notably higher at roadside air-monitoring stations [48].

### Highlights of this study

We conducted a survey of respiratory symptoms among children and measured the air pollution levels in Ulaanbaatar, including in a suburban area. No previous studies have examined the prevalence of respiratory symptoms among children in suburban areas. Furthermore, outdoor air pollutants were being measured at ambient air quality monitoring stations in the urban areas of Ulaanbaatar. However, such systems have not been installed in the suburban areas surrounding the city. Moreover, traffic volume was assessed in a suburban area for the first time in this study.

### Limitations of this study

In this study, we only measured the levels of NO_2_ and SO_2._ The obtained SO_2_ concentrations were lower than those obtained via national air quality monitoring because they were measured at sites located within 200 m of major roads. Also, this study did not measure the levels of particulate matter, and the personal exposure levels of each subject were not evaluated. In addition, this study did not use continuous monitoring data, and the measurement of air pollutants was conducted over a short period of time. The concentrations of air toxins, such as diesel exhaust particles or surrogates, such as black carbon or soot, should be more widely monitored.

## Conclusions

This study investigated the effects of short-term outdoor air pollution exposure on the respiratory symptoms of children in Ulaanbaatar. The prevalence of persistent cough symptom was high among schoolchildren in urban and suburban districts of Ulaanbaatar. The outdoor SO_2_ concentration was associated with the persistent cough symptom, and the NO_2_ concentration was associated with the current wheezing symptom among children. We also found that living near a major road results in children being exposed to traffic-related air pollution, which could aid future epidemiological studies. A study of air pollution control in suburban areas is necessary to promote public health.

## Supplementary information

**Additional file 1.** **Table S1.** Definition of respiratory symptoms in questionnaire. **Figure S1.** The mapping of residential address. **Table S2.** Statistically significant differences among the SO_2_ concentration in study areas. **Table S3.** Statistically significant differences among the NO_2_ concentration in study areas. **Table S4.** Odds ratios of respiratory symptoms in realtion to personal and environmental factors. **Table S5.** Comparison of prevalence of respiratory symptoms among children by residential areas

## Data Availability

The datasets used and/or analyzed during the current study are available from the corresponding author on reasonable request.

## Abbreviations

ATS-DLD: American Thoracic Society Division of Lung Disease
CI: Confidence interval
NO_2_: Nitrogen dioxide
OR: Odds ratio
SO_2_: Sulfur dioxide
