# Supplementary material for: Coal burning-derived SO2 and traffic-derived NO2 are associated with persistent cough and current wheezing symptoms among schoolchildren in Ulaanbaatar, Mongolia
Source: Environ Health Prev Med. 2019 Nov 27;24:66. doi: 10.1186/s12199-019-0817-5 (PMC6882210; doi:10.1186/s12199-019-0817-5)
Supplement: Supplementary file 1 — Additional file 1. Table S1. Definition of respiratory symptoms in questionnaire. Figure S1. The mapping of residential address. Table S2. Statistically significant differences among the SO2 concentration in study areas. Table S3. Statistically significant differences among the NO2 concentration in study areas. Table S4. Odds ratios of respiratory symptoms in realtion to personal and environmental factors. Table S5. Comparison of prevalence of respiratory symptoms among children by residential areas [file 12199_2019_817_MOESM1_ESM.docx]

Additional file 1

Table S1. **Definition of respiratory symptoms in questionnaire**

| Respiratory symptom | Definition |
| --- | --- |
| Persistent cough | cough on most days (four or more days per week) for at least three months a year either with or without cold |
| Persistent phlegm | congested or brought up phlegm, mucus from the chest on most days (four or more days per week) for at least three months a year either with or without cold |
| Wheezing | chest ever sound wheezy or whistling, including times when he/she a has cold and two or more such episodes in the past two years |
| Asthmatic-like symptom | have experience an attack of wheezing or whistling and suddenly caused to be short of breath, had an attack like that two or more times, have been diagnosed with childhood asthma by a doctor, wheezing or whistling could be heard during an attack, short of breath and wheezing during an attack, experienced such an attack or have received treatment for asthma in the past two year. |

Figure S1


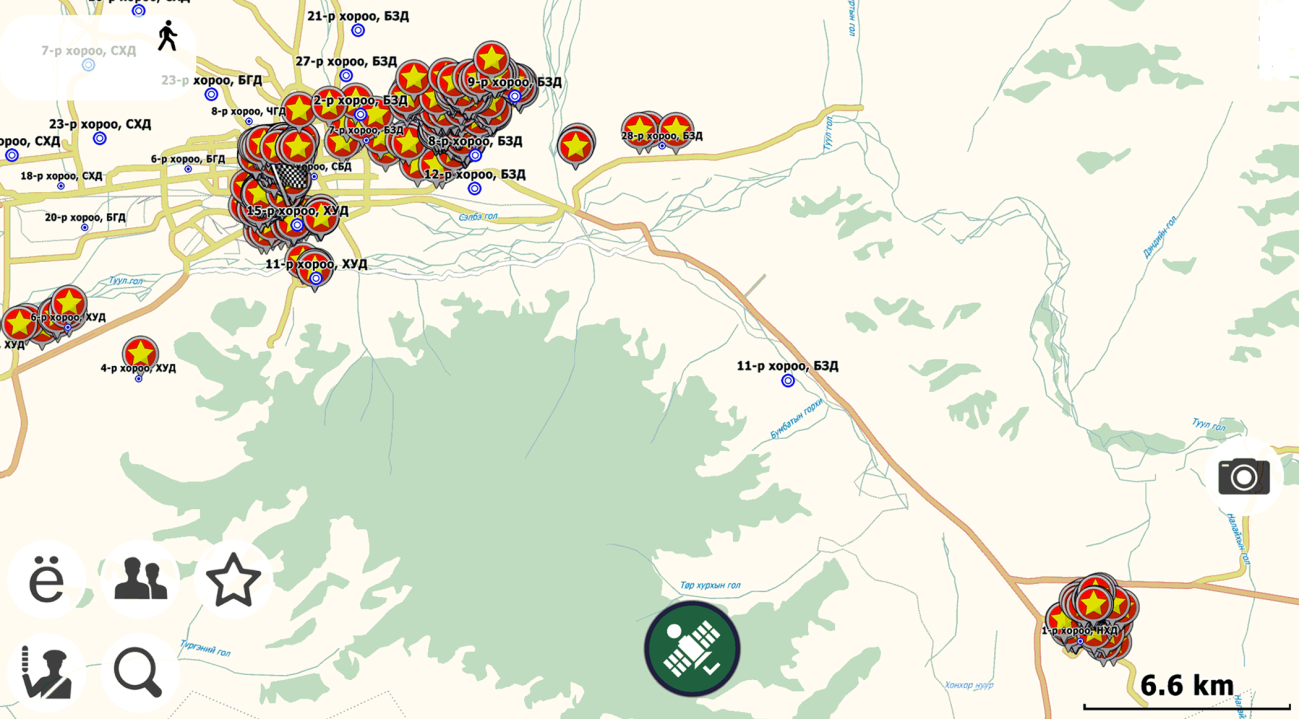


Table S2 Statistically significant differences among the SO_2_ concentration in study areas

| District | winter (Jan2015,Feb2016) | spring  (April 2015,2016) | summer (Aug2015,2016) | autumn  (Nov 2015,2016) |
| --- | --- | --- | --- | --- |
|  | SO_2_ mean±SD ppb (min-max) | | | |
| A | 14.3±9.0 (6.0-36.0) | 2.0±1.5 (0.0-5.2) | 0.8±.0.7 (0.0-1.8) | 5.0±2.5 (1.4-9.5) |
| B | 12.3±10.2 (4.0-39.0) | 3.0±1.9 (0.0-6.7) | 1.2±0.9 (0.0-3.1) | 2.9±1.7 (0.5-7.0) |
| C | 21.9±17.6 (8.0.-63.0) | 2.6±1.8 (0.2-6.7) | 0.9±0.7 (0.0-2.8) | 9.6±8.3 (0.5-30.0) |
| D | 19.6±11.6 (5.0-46.0) | 2.1±3.2 (0.3-13.7) | 0.7±0.8 (0.00-0.8) | 8.0±4.6 (0.9-15.7) |
| t test | p value | | | |
| A vs B | 0.295 | 0.016 | 0.124 | 0.000 |
| A vs C | 0.000 | 0.267 | 0.478 | 0.019 |
| A vs D | 0.006 | 0.892 | 0.879 | 0.003 |
| B vs C | 0.000 | 0.327 | 0.179 | 0.004 |
| B vs D | 0.003 | 0.279 | 0.139 | 0.000 |
| C vs D | 0.000 | 0.639 | 0.482 | 0.392 |

Table S3. Statistically significant differences among the NO_2_ concentration in study areas

| District | winter (Jan2015,Feb2016) | spring (April 2015,2016) | summer (Aug2015,2016) | autumn (Nov 2015,2016) |
| --- | --- | --- | --- | --- |
|  | NO_2_ mean±SD ppb (min-max) | | | |
| A | 54.6±17.1 (36.0-86.0) | 20.0±3.9 (14.0-27.0) | 16.5±3.8 (12.0-25.0) | 28.7±8.5 (19.0-50.0) |
| B | 48.1±15.1 (22.0-74.0) | 21.6±5.4 (13.0-33.0) | 16.5±6.0 (7.0-28.0) | 24.7±9.2 (12.0-48.0) |
| C | 56.3±21.4 (24.0-99.0) | 17.5±7.3 (10.0-42.0) | 16.9±7.4 (4.0-31.0) | 28.7±8.0 (20.0-49.0) |
| D | 40.5±14.1 (24.0-68.0) | 9.1±1.9 (5.0-13.0) | 6.1±1.8 (3.00-8.0) | 17.4.0±8.1 (8.0-41.0) |
| t test | p value | | | |
| A vs B | 0.014 | 0.131 | 1.000 | 0.000 |
| A vs C | 0.560 | 0.110 | 0.770 | 0.972 |
| A vs D | 0.000 | 0.000 | 0.000 | 0.000 |
| B vs C | 0.005 | 0.012 | 0.788 | 0.005 |
| B vs D | 0.004 | 0.000 | 0.000 | 0.000 |
| C vs D | 0.000 | 0.000 | 0.000 | 0.000 |

Table S4. Odds ratios of respiratory symptoms in realtion to personal and environmental factors

| Confounding factors | persistent  cough | persistent phlegm | current wheezing |
| --- | --- | --- | --- |
| age  (9-12 yrs old (ref : 6-8 yrs) | 1.05 (0.80, 1.38) | 0.85 (0.63, 1.16) | 1.73 (0.98, 3.07) |
| sex  (male ref : female) | 0.94 (0.72, 1.24) | 1.09 (0.80, 1.47) | 0.75 (0.44, 1.28) |
| feeding method in infancy  ( bottle milk vs breast feeding) | 1.03 ( 0.72, 1.45) | 0.90 (0.61, 1.34) | **2.04** (1.14, 3.66) |
| history of respiratory diseases before 2 yrs old (yes vs,no) | 1.01 (0.71, 1.42) | **1.58** (1.10, 2.25) | 1.65 (0.89, 3.03) |
| history of diagnosed asthma  (yes vs,no) | **3.83** (1.64-.8.97) | **2.59** (1.14-5.88) | 1.11 (0.32-3.91) |
| history of allergic diseases  (yes vs,no) | 1.20 (0.82, 1.75) | 0.96 (0.63, 1.46) | 1.37 (0.72, 2.60) |
| history of pneumonia  (yes vs, no) | 1.67 (0.93, 3.00) | **1.90** (1.07, 3.36) | **4.01** (1.87, 8.60) |
| parental smoking habit  ( ref : other than parents) | 1.25 (0.95, 1.65) | 0.97 (0.71, 1.32) | 1.46 (0.86, 2.50) |
| history of residence year  (more 3 yrs ref: less than) | 1.14 (0.85, 1.53) | 0.66 (0.48, 0.90) | 0.99 (0.57, 1.71) |
| Heating type ( coal using ref :centralized heating) | **1.81** (1.24, 2.62) | 1.38 (0.90, 2.12) | 0.89(0.40, 1.99) |
| Distance from main road  ( <100 m ref : > 100 m) | **1.86** (1.37, 2.51) | 1.29 (0.93, 1.79) | 1.28 (0.73, 2.22) |

*^a^Data are presented as odds ratio (95%,confidence interval)*

*^b^ bolded values indicate statistical significant : p <0.05*

*^c^ mean concentrations for outdoor SO_2_ of each district 5.2 ppb, 4.4 ppb, 10.1 ppb, and 7.1 ppb, for NO_2_ -30.0 ppb, 27.7 ppb, 30;.0 ppb and 18.2 ppb, respectively. And traffic volume was calculated as a value per thousand vehicles a day of each district: 16.84, 16.99, 19.47and 7.19, respectively.*

**Table S5** Comparison of prevalence of respiratory symptoms among children by residential areas

| Residential area | Apartment (A,B) | | Ger (C,D) | |  |
| --- | --- | --- | --- | --- | --- |
| Respiratory symptoms | n | % | n | % | p |
| persistent cough | 110 | 21.6 | 206 | 34.4 | 0.000 |
| persistent phlegm | 100 | 19.6 | 124 | 20.7 | 0.674 |
| current wheezing | 36 | 7.1 | 28 | 4.8 | 0.815 |
| asthma like symptom | 4 | 0.8 | 4 | 0.7 | 0.087 |
